# Achievements of the past and Hopes for the Future

**Published:** 1921-01-22

**Authors:** 


### THE LEPER PROBLEM IN INDIA.

### Achievements of the Past and Hopes for the Future.

In the year 1889 Father Damien died from leprosy, and his death directed world-wide attention to the disease and its victims. The interest led to the appointment of a National Leprosy Commission, which visited India and published a report which created a good deal of interest.

Chiefly as the result of this report, the Lepers' Act for the segregation and medical treatment of pauper lepers was passed in 1898. The Act was permissive, and has been of little service. The fundamental weakness of the Act was the definition of a leper. A leper was defined as one in whom the process of ulceration has commenced, and it was necessary for the police to take pauper lepers whom it was desired to segregate before a magistrate. The police did not like the task, and the Act was a dead letter. Under the Act Government leper asylums were inaugurated, but institutions of this kind had already been going for some years previously, under the auspices of the Mission to Lepers, which was founded in 1874 by a group of Irish and Scotch philanthropists.

### The Work of the Mission.

The work of this Mission has been steadily progressing, but, largely due to the work of Sir Leonard Rogers and the untiring labours of a very energetic secretary, public attention in India has been specially directed to the disease during the past few months. The secretary has been giving eloquent addresses and lantern lectures all over India, and under the auspices of the Mission to Lepers a conference of asylum superintendents and others was held recently in Calcutta. The conference was attended by the Sanitary Commissioner, with the Government of India and other official delegates, and papers were read on almost every aspect of the leper problem, from the therapeutics of the latest drugs to minor administrative details, such as the organisation and care of gardens and dairies in leper asylums. The report of the conference is now to hand, and is a most illuminating and well-illustrated document, which reflects great credit on the secretary to the conference and the Society which he serves. The attention of the reader is arrested at the outset by an excellent graph, which shows that the estimated number of lepers in India is 150,000, and that only 8,850 are in asylums! Even officials who have lived for years in India will be impressed by the extent to which its population is infected, and people who do not know India and her disregard of hygienic laws will be struck by an excellent photograph, which illustrates a colony of lepers which lives in the centre of Calcutta and moves freely about amongst the untainted community.

### A Special Committee.

The conference adopted the unanimous Findings of a Special Medical Sub-Committee, which may be summarised as follows:

(1) Leprosy is contagious through the escape of the causative bacillus in nasal discharges or open sores. The incubation period is eight to fourteen years. The bacillus has not been cultivated *in vitreo*, and laboratory experiments have been hampered in consequence.

(2) The disease is not hereditary, but the children of lepers are specially susceptible to the disease.

(3) Segregation is the most effective measure for reducing the prevalence of the disease, and the grave danger to the community of unrestricted association with lepers.

(4) In view of the fecundity of lepers, especially of females, and the excessive danger of contagion to the children of lepers, which play a great part in maintaining the prevalence of the disease, the separation of the sexes is desirable. Married lepers should only be allowed to live together on the understanding that any children born to them shall be separated from their infected parents at the earliest possible age. Married patients presenting good prospects of recovery should always be separated to eliminate the risk of re-infection during treatment.

(5) Treatment with the salts of fatty acids has been tested by fourteen medical officers in leper asylums throughout India, with the result that 72 per cent. showed marked improvement, in spite of the fact that most of the cases were advanced and the period of treatment short. It is impossible in the present stage of leper therapy to say more than that the prospects of cure in early cases are distinctly good. The pauper leper is the chief menace to the public health of India, and the Medical Sub-Committee urged the necessity for the establishment of settlements for this class of case

### The Results of Treatment.

The seven medical papers read before the Conference by Sir Leonard Rogers, and Doctors Muir, Neve, Parker, Chatterji and Joshee should be consulted by all who wish to gain an insight into the modern treatment of the disease. Sir Leonard Rogers, Dr. Chatterji, and Dr. Neve devoted themselves to general details of treatment by the salts of fatty acids, whilst Dr. Muir dealt with the after-treatment of leprosy cases, and Dr. Joshee with the treatment of ulcers. Dr. Parker gave an interesting account of Heiser's method of treatment which had been carried out in her asylum at Manamadura, in the Madras Presidency, since 1916.

We would remind our readers that this treatment consists of deep intra-muscular injections in increasing doses of a mixture of resorcin, chaulmoogra oil, and camphorated oil, in the following proportions:

| | |
|---|---|
| Resorcin, 4 grm.<br>Ol. chaulmoogra, 60 c.c.<br>,, camph., 60 c.c. | Minimum dose, 1 c.c.<br>Maximum dose, 10 c.c. |

Nearly all cases treated by Heiser's method improved, whilst in eight cases all symptoms disappeared. Of the cured cases, six were anæsthetic, one tubercular, and one mixed.

### Administrative Questions.

The administrative side of work amongst lepers naturally vied with the purely scientific discussions in the work of the Conference, and a reunion of such a large number of leper asylum superintendents must be productive of much good. The Sanitary Commissioner with the Government of India dealt with sites, drainage, and water supply of leper institutions, and the Rev. P. A. Penner and G. E. Hicks with their construction.

The outstanding features of the Sanitary Commissioner's paper were (1) that, given a clear water, any standard of purity can be obtained by the use of chlorine or one of its compounds, and (2) that in small institutions, provided the soil is permeable, sullage water can be disposed of in filter trenches. He referred the

**The Leper Problem in India—(*continued*).**

members of the Conference to Dr. Vivian Poore's books on rural sanitation, and concluded that "it is possible to work an institution in a rural area without any great expenditure of money. The essentials are preliminary advice from experts and the training in small details of such members of the staff of the institution as are gifted with ordinary intelligence." The dominant note of the speakers on asylum construction was that the leper ward should be constructed so as to be a comfortable residence for the leper, and *not* a barracks; and one speaker urged that the name asylum should be changed to "Home."

One of the most interesting and exhaustive papers read before the Conference was by Lieut.-Colonel Robert McCarrison on "The Diet in Leper Asylums." The writer emphasised and explained the necessity in leper dietary not only of food, but of the accessory food factors or vitamines. Colonel McCarrison dealt with the dietaries of thirty-three asylums, and declared that "few are sufficiently well-balanced to maintain a healthy individual in health, and fewer still are of a composition which will enable the leprous tissues to regenerate." He pointed out that lepers require a proportion of their protein in a readily assimilable form—*i.e.*, in a form in which it is readily "built into" the tissues. In Colonel McCarrison's opinion, the best way to secure this is to give each leper two raw eggs per diem. He insisted on the need for quantities of water, preferably hot, to flush out the glands and keep them "sweet," as lepers have more waste material to get rid of than healthy folk. He regarded potatoes and tomatoes as essential constituents of leper diet, and concluded by stating "in general terms the dietaries used in the majority of leper asylums are seriously deficient in fats and in vitamines," often deficient in the proper kind of protein or building stones, and frequently disproportionately rich in carbohydrates. His solution of the difficulties with regard to diet is to make each asylum self-supporting with respect to dairy produce and to eggs and to fruit and vegetables, reminding us again that no form of medicinal treatment of leprosy can be successful without the provision in the food of those materials on which the growth and repair of the tissues are dependent. He added a specimen dietary for an adult male leper, living in a fairly temperate climate and doing moderately hard work, which was composed as follows: Atta (coarse wheaten flour), ½ lb.; rice, ¾ lb.; lentils, 4 oz.; nuts, 1 oz.; butter, 2 oz.; eggs, two; potatoes and tomatoes, of each 4 oz.; sugar, 1 oz.; hot water, 2 pints; buttermilk, 1½ pint; spices and salt as required, and vegetables and fruit as much as possible.

The Conference focussed public attention, and it is interesting to know that since it was held the Leper Amendment Act of 1920 has become law. This Act gives powers to local Governments to make provision for the segregation of pauper lepers in asylums and in leper settlements, and defines as a leper a person suffering from any variety of leprosy. The Conference was unanimously of the opinion that the disease of leprosy could be stamped out in India as it has been in these islands, if *all* lepers were segregated.

This does not appear practicable at present, but the Government of India is to be congratulated on having taken the first step in this direction by passing legislation for the segregation of all pauper lepers. Thanks to the efforts of the Mission to Lepers the public conscience in India has been quickened, and the Mission, which was, as we have said, inaugurated and has been maintained for nearly half a century by British philanthropists, is now receiving large financial and—what is almost more important—cordial moral support from Indians themselves of every race and creed.

What the future holds hidden in its bosom for the nations of the world none can predict. We believe, however, that the Mission is justified in its belief that "for the lepers the rainbow of hope has at last shone forth in their dark and gloomy sky."

